# Diurnal Nest Site Selection and Thermal Buffering Capacity of Shelters for Rufous Bettongs in a Grazed Savanna Woodland

**DOI:** 10.1002/ece3.74592

**Published:** 2026-11-02

**Authors:** Eric J. Nordberg, Natasha Ryan, Heather Neilly, Lin Schwarzkopf

**Affiliations:** ^1^ School of Environmental and Rural Science, University of New England Armidale New South Wales Australia; ^2^ College of Science and Engineering, James Cook University Townsville Queensland Australia; ^3^ Future Regions Research Centre, Federation University Mt Helen Victoria Australia

**Keywords:** *Aepyprymnus rufescens*, agriculture, habitat selection, microclimate, potoroidae, thermoregulation

## Abstract

Small‐ and medium‐sized mammals are vulnerable to temperature extremes due to their high surface‐area‐to‐volume ratios and elevated metabolic rates. Shelter sites that buffer ambient temperatures are critical for thermoregulation; however, shelter resources may be limited in agricultural landscapes. We examined shelter site selection and the thermal buffering capacity of rufous bettong ( *Aepyprymnus rufescens* ) shelters in a grazed tropical savanna woodland in Queensland, Australia. We radio‐tracked 15 rufous bettongs (nine females, six males) across five grazing strategies at Wambiana Station (2020–2021) to locate diurnal shelters. We assessed fine‐scale habitat composition at 133 shelter sites and 224 paired random locations using a MANOVA and NMDS ordination. We deployed iButton data loggers to record hourly temperatures within shelters and at paired ground‐level points 2 m away across wet and dry seasons, isolating the thermal effect of the constructed shelter at the locations the animals selected. Rufous bettongs selected shelter sites characterized by thorny shrubs (*Carissa ovata*), logs, and termite mounds. Grazing intensity did not significantly affect shelter site selection, though bettongs tended to avoid heavily grazed paddocks. Daytime shelter temperatures (0600–1800 h) were substantially less variable than those at random sites, with thermal buffering most pronounced during the day (0900–1700 h), when random sites exhibited 2–3 times greater temperature variability than bettong shelters. Shelters provided critical thermal refugia, buffering daily temperature extremes by over 20°C in a hot, seasonally variable environment. Retention of dense shrub thickets ( *C. ovata* ), logs, and adequate grass cover should be prioritised in grazing landscapes to support rufous bettong persistence under current and future climate conditions.

## Graphical abstract

Small mammals in hot climates depend on shelter to survive high daytime temperatures. We show that rufous bettongs construct grass nests that cut daytime temperature extremes by more than 20°C, and that this buffering holds wherever they build, including in the open. Retaining the shrubs, logs, and grass they build with is therefore central to their persistence in grazed rangelands under a warming climate.

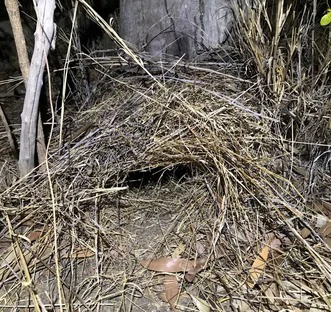

## 1 Introduction

Shelter sites protect animals from predators and weather extremes, and are critical for survival (Fisher 2000; MacGregor et al. 2015; Waudby and Petit 2017). They can take many forms depending on the species, habitat, and local climatic conditions. Some animals use existing structures as shelter, such as logs, tree hollows, and caves (Wilkinson et al. 1998; Gibbons and Lindenmayer 2002; Cockle et al. 2011), while others create shelters, like burrows and nests (Haussmann 2017; Linley et al. 2024; Machicote et al. 2004; Qiu and Schradin 2024).

Small‐ and medium‐sized mammals (e.g., < 5 kg) are particularly vulnerable to temperature extremes because their high surface‐area‐to‐volume ratios and metabolic rates (McNab 1983) lead to increased heat loss during cold periods and heat gain during warm periods (Canals et al. 1989; Geiser 2004; Speakman and Król 2010). Shelters can reduce heat loss in cold conditions (Walsberg 1986) and mitigate heat stress in warm conditions (Denny and Dawson 1975). Shelter location, structure, and composition critically influence thermal buffering capacity—the ability to reduce exposure to thermal extremes. Subterranean burrows strongly attenuate temperature fluctuations (Mathias et al. 2004), whereas surface shelters, including logs and tree hollows, typically track ambient conditions more closely but still provide thermal buffering (Brice et al. 2002; Rowland et al. 2017; Griffiths et al. 2018). Importantly, shelter microclimates can differ significantly from external conditions, providing thermal refuge from extremes and reducing heat loss through radiative shielding (Beck and Jennings 2003; Howarth et al. 2025; Bhattacharyya et al. 2015).

In agricultural systems, shelter availability may be compromised through habitat modification such as clearing, which can reduce key resources including leaf litter, fallen logs, standing dead trees, and hollow‐bearing trees (Fischer et al. 2010; Neilly et al. 2016). Livestock grazing can further modify understorey vegetation structure (Fleischner 1994), reducing ground cover and structural complexity that many terrestrial wildlife species depend on for shelter and predator avoidance (Neilly, Nordberg, et al. 2018; Neilly and Schwarzkopf 2017, 2019). However, grazing landscapes can be compatible with biodiversity conservation. When managed sustainably, grazed systems can maintain habitat heterogeneity and structural complexity that support diverse wildlife communities (Neilly et al. 2016; Krausman et al. 2009; Barroso and Gortázar 2024). Implementing grazing strategies that balance agricultural productivity with the retention of critical habitat features, such as maintaining vegetation cover, preserving woody debris, and managing stocking rates to prevent overgrazing, may prevent degradation of shelter habitats (Eldridge et al. 2016; Neilly, O'Reagain, et al. 2018). Understanding the shelter ecology of wildlife species that persist in grazed landscapes is therefore directly relevant to informing sustainable land management.

The rufous bettong ( *Aepyprymnus rufescens* ) is the largest rat‐kangaroo (Potoroidae), restricted to coastal and sub‐coastal eastern Australia from northern Queensland to the New South Wales–Victoria border (Van Dyck and Strahan 2008). Rufous bettongs are ecosystem engineers, engaging in soil‐disturbing activities which enhance soil function and increase soil productivity (Neilly et al. in review). Unlike many sympatric small‐ and medium‐sized terrestrial mammals, the rufous bettong has proven somewhat resilient to land use changes and is the only potoroo or bettong persisting in cattle‐grazing landscapes (Short 1998; Ryan 2023). In grazed savanna woodlands in north Queensland, rufous bettongs are widespread but avoid areas of highest cattle utilisation and prefer areas with higher grass cover (Neilly and Schwarzkopf 2018). The characteristics of shelter sites used by rufous bettongs may be key to understanding their persistence in these modified landscapes.

Rufous bettongs rest during the day in shallow, dome‐shaped nests constructed from grass and other vegetation, typically at the base of grass tussocks or shrubs that provide concealment and protection (Schlager 1982). Unlike other bettongs that use soil burrows or shelters deep within grass tussocks, rufous bettong shelters may lack capacity for significant thermal buffering due to their shallow construction. The thermal buffering provided by shelter sites may therefore depend on shelter structure or the structural complexity of surrounding vegetation. Given their role as ecosystem engineers, understanding the shelter requirements that enable rufous bettongs to persist in grazed systems has implications for both species conservation and broader ecosystem function.

Here, we examined shelter site selection of rufous bettongs under a range of grazing strategies in a savanna woodland in north Queensland, Australia, and investigated the thermal buffering capacity of their shelters. We aimed to: (1) characterize fine‐scale shelter site selection in relation to vegetation cover and structural complexity; (2) examine whether landscape‐scale shelter site selection differs among grazing strategies; and (3) quantify the thermal buffering provided by the constructed shelter by comparing temperatures inside shelters with those at paired points 2 m away at the same locations. Aims (1) and (2) establish where bettongs choose to build shelters; aim (3) then asks what building a shelter achieves thermally at those locations. The thermal comparison is therefore between a shelter and the same location without a shelter, and is not intended to compare shelters with alternative resting sites elsewhere in the landscape.

## 2 Methods

### 2.1 Study Site

This study was conducted at the Wambiana grazing trial (Queensland Department of Agriculture and Fisheries; O'Reagain et al. 2018), located approximately 70 km southwest of Charters Towers on Wambiana Cattle Station. This savanna woodland site is characterized by distinct wet and dry seasons. Five grazing strategies were trialled (Table 1), with two replicates of each across 10 paddocks (97–117 ha). Dominant vegetation types include Reid River box (*Eucalyptus brownii*) and Silver‐leaf ironbark ( *Eucalyptus melanophloia* ), with a narrow band of brigalow (*Acacia harpophylla*) separating them. Paddocks were designed to contain equal areas of each vegetation type (approx. two‐thirds Reid River box at the northern end, one‐third silver‐leaf ironbark at the southern end, and a strip of brigalow in‐between; O'Reagain et al. 2018). Neilly and Schwarzkopf (2018) estimated that approximately 56 ± 5 (SE) rufous bettongs inhabited the Wambiana grazing trial during 2014–2015 surveys, with estimated densities of 0.01–0.03 individuals/ha.

**TABLE 1:** Grazing strategies trialled at the Wambiana Grazing Trial, Queensland, Australia.

Grazing strategy	Abbreviation	Stocking rate (ha/AE)	Description
Heavy stocking rate	HSR	4	Continuous grazing at ~twice LTCC
Moderate stocking rate	MSR	8	Continuous grazing at LTCC
Rotational spelling	R.SPELL	8	Rotated among 6 sub‐paddocks with wet season spelling
Flexible + wet season spelling	FLEX.S	4–12	Stocking adjusted to forage availability; rotated among 6 sub‐paddocks
Flexible (no spelling)	FLEX	4–12	Stocking adjusted to forage availability; no spelling

Abbreviations: AE = animal equivalent (450 kg steer), LTCC = long‐term carrying capacity.

### 2.2 Landscape‐Scale Shelter Site Selection

The locations of diurnal shelter sites were collected by attaching LiteTrack 20 VHF radio‐tracking collars (Lotek Wireless, Newmarket, Ontario, Canada) to 15 rufous bettongs (nine females, six males). The female‐biased sex ratio among collared individuals was a product of capture success rather than by design. To attach the transmitters, individuals were captured using cage traps baited with standard bait (balls of peanut butter, oats, and vanilla essence). Cage traps were deployed across all 10 paddocks and checked at dawn each morning. The sample size of 15 individuals was constrained by the cost of VHF collars. Trapping success was highest in the northern ends of each paddock, which were dominated by Reid River box (*Eucalyptus brownii*) woodland. Paddocks were separated by three‐strand electric fencing designed to contain cattle, and bettongs could easily pass beneath the bottom wire. Given the small spatial scale of individual paddocks relative to bettong home ranges, all tracked individuals had unrestricted access to all grazing treatments regardless of initial capture location.

Collared individuals were tracked using a three‐element Yagi antenna and a Telonics R‐1000 telemetry receiver between January 2020 and May 2021. Animals were relocated during the day 1–2 times per fortnight to identify diurnal shelter sites by homing in on the VHF signal. Once the signal indicated the animal was stationary within a shelter, a waypoint was recorded on a handheld Garmin GPS.

### 2.3 Landscape‐Scale Statistical Analyses

Location data were filtered to include only the five experimental grazing strategies (FLEX.S, FLEX, R.SPELL, MSR, HSR; Table 1) implemented within the Wambiana grazing trial. Animals occasionally moved onto adjacent private property (outside the managed paddocks), which was not subject to the same standardized stocking rate management. To ensure comparability across the five managed grazing strategies, all locations recorded on private property were excluded from landscape‐scale habitat selection analyses.

We assessed landscape‐scale shelter site selection using a WIDES Type II Manly selectivity measure (R package “adehabitatHS” v0.3.15; Calenge 2011), which tests whether individuals select shelter sites in proportion to what is available in the landscape. We ran a single pooled analysis across all 15 individuals. Because the adehabitatHS package does not support covariates within the Manly Type II selection ratio framework, we compared individual selection ratios between sexes post hoc using Wilcoxon rank‐sum tests with Bonferroni correction. With nine females and six males, statistical power to detect sex differences was limited.

GPS waypoints from each individual's shelter sites were categorised as “used” habitat data. Habitat availability was assessed on a per‐event basis rather than a per‐structure basis. Each time an animal was tracked to a shelter, a new paired random point was generated at a random distance (within 50 m) and random bearing from the shelter site, without regard to whether the location could support a shelter. Because individuals sometimes re‐used shelters, the total number of random points assessed in our study exceeded the number of unique shelter locations.

### 2.4 Fine‐Scale Shelter Site Selection

We conducted habitat assessments at each unique shelter site and at the paired random location 50 m away in a randomly generated direction. Habitat assessments were completed at 133 of the 136 unique shelter sites located. Within a 1 × 1 m quadrat, we recorded the proportion of fine woody debris, bare ground, grass, leaf litter, shrubs/saplings, *Carissa ovata*, logs, trees, and termite mounds. Given that rufous bettong shelters are shallow, dome‐shaped structures typically < 50 cm in diameter, the quadrat captured the immediate microhabitat surrounding the shelter in addition to the shelter structure itself. Canopy cover was estimated from an upward‐pointing, vertically oriented photograph taken 1 m above each site.

### 2.5 Fine‐Scale Statistical Analyses

We compared structural composition between shelter and random sites using a one‐way MANOVA with Pillai's trace, as it is robust to unequal sample sizes and departures from multivariate normality, with site type (shelter vs. random) as a fixed factor and 10 structural variables as response variables: *Carissa ovata* cover, leaf litter, bare ground, grass, logs, fine woody debris, trees, saplings/shrubs, termite mounds, and canopy cover. The MANOVA was based on the 133 unique shelter sites at which habitat was assessed; habitat was assessed once per unique location, so shelter reuse did not introduce pseudoreplication. The 224 random sites reflect the per‐event paired design described above. Following a significant MANOVA, we examined univariate ANOVAs to determine which variables differed between site types. We report partial eta‐squared (*η* ^2^_p_) effect sizes alongside F‐statistics to convey the magnitude of habitat differences. We visualised multivariate differences using non‐metric multidimensional scaling (NMDS) with Bray–Curtis dissimilarity (*k* = 2 dimensions, 100 iterations) using the vegan package in R (Oksanen et al. 2024).

### 2.6 Shelter Site Temperature

We recorded shelter site temperatures hourly using Thermochron iButton data loggers sealed in a waterproof wax coating (Roznik and Alford 2012). At each site, one iButton was placed within an unoccupied shelter and one at a paired random point within 2 m, both secured to the ground in neutral‐colored calico bags using aluminium garden pegs. Because each paired point lay within 2 m of the shelter, both loggers shared the same overstorey, soil, aspect, paddock and grazing treatment, and the pair therefore differed principally in the presence or absence of the constructed shelter. This comparison quantifies the thermal effect of shelter construction at the location the animal selected. These paired thermal points are distinct from the paired random points used in the fine‐scale habitat assessments, which lay up to 50 m away and characterize habitat availability rather than thermal conditions. They are not intended to represent alternative resting sites that a bettong might otherwise have chosen. iButtons were deployed for 3–4 months per season. A total of 40 iButtons (20 shelter, 20 random) were deployed during the dry season (July–October 2020) and again in the wet season (December 2020–March 2021). Shelters were confirmed to be unoccupied at the time of iButton placement; however, we cannot exclude the possibility that bettongs returned to and occupied some shelters during the recording period. An inspection of the thermal data revealed no instances of suspiciously constant daytime temperatures (similar to bettong body temperatures) that would indicate the presence of an animal influencing the shelter site temperature.

### 2.7 Shelter Site Temperature Statistical Analyses

As rufous bettongs are nocturnal and occupy shelters during daylight hours, we restricted the thermal analysis to daytime hours (0600–1800 h). We calculated the coefficient of variation (CV) of hourly temperatures at shelter and random sites. We also calculated the mean hourly Δ*T* (unsheltered minus shelter temperature) across loggers, with 95% confidence intervals computed across loggers as the unit of replication, to provide a direct measure of thermal buffering at ecologically relevant times of day. To test whether buffering depended on how exposed a shelter was, we also compared the daytime maximum temperature and CV per logger between shelter and paired unsheltered loggers using Mann–Whitney *U* tests, treating the daytime maximum temperature inside a shelter as an index of its solar exposure. We analyzed each season separately using linear mixed‐effects models (R package “lme4” v1.1–26; Bates et al. 2015) with location (shelter vs. random), hour, and their interaction as fixed effects, and iButton ID as a random effect. The significance of fixed effects was assessed using Type III Wald chi‐square tests (R package “car” v3.1–0; Fox and Weisberg 2019), with sum‐to‐zero contrasts applied to ensure valid interpretation of main effects in the presence of the interaction. We report marginal and conditional *R* ^2^ values using the “piecewiseSEM” package (Lefcheck et al. 2018) and assessed model fit using the “DHARMa” package (Hartig 2022). Model assumptions were checked by an inspection of residual plots. All statistical analyses are reported as mean ± SD and were conducted in R (v4.4.1; R Core Team 2024).

## 3 Results

### 3.1 Shelter Sites—General Description

Rufous bettong shelters consisted of shallow pits (approximately 10–20 cm deep, 30 cm diameter) covered by a thick layer of grass forming an enclosed ‘roof’ (Figure 1). Grass is collected and carried in the animal's prehensile tail (Schlager 1982; Supporting Information—Video S1). Rufous bettongs did not actively close the entrance of their shelters; the grass covering provides concealment and some thermal insulation but does not form a sealed enclosure. Individuals maintained a network of shelters, sometimes reusing sites weeks later (Figure 2). Animals were relocated 1–2 times per fortnight, so consecutive‐day shelter use could not be assessed directly; however, individuals were rarely recorded at the same shelter on successive tracking events. Shelters typically contained one animal, except for one female observed sharing with her joey throughout the study.

**FIGURE 1:**
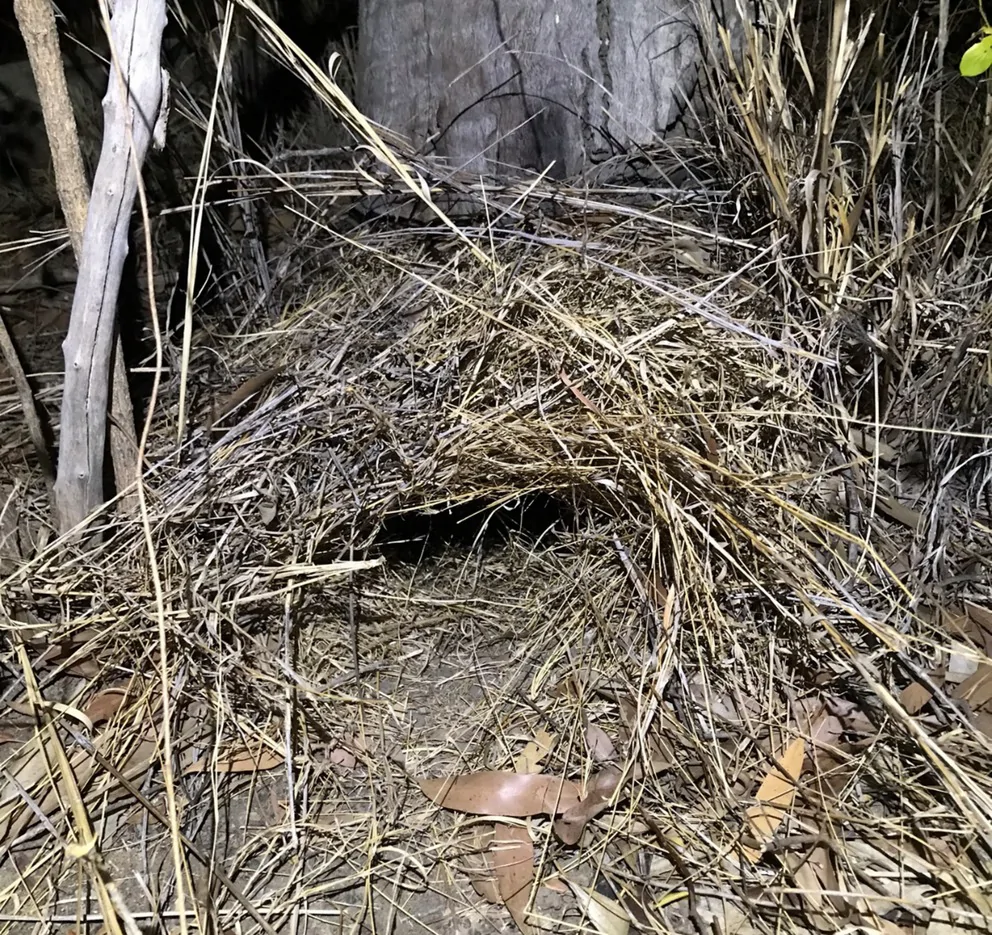
Rufous bettong shelter site at Wambiana Station, showing the grass “roof” covering the excavated pit (Photograph: E. Nordberg 2020).

**FIGURE 2:**
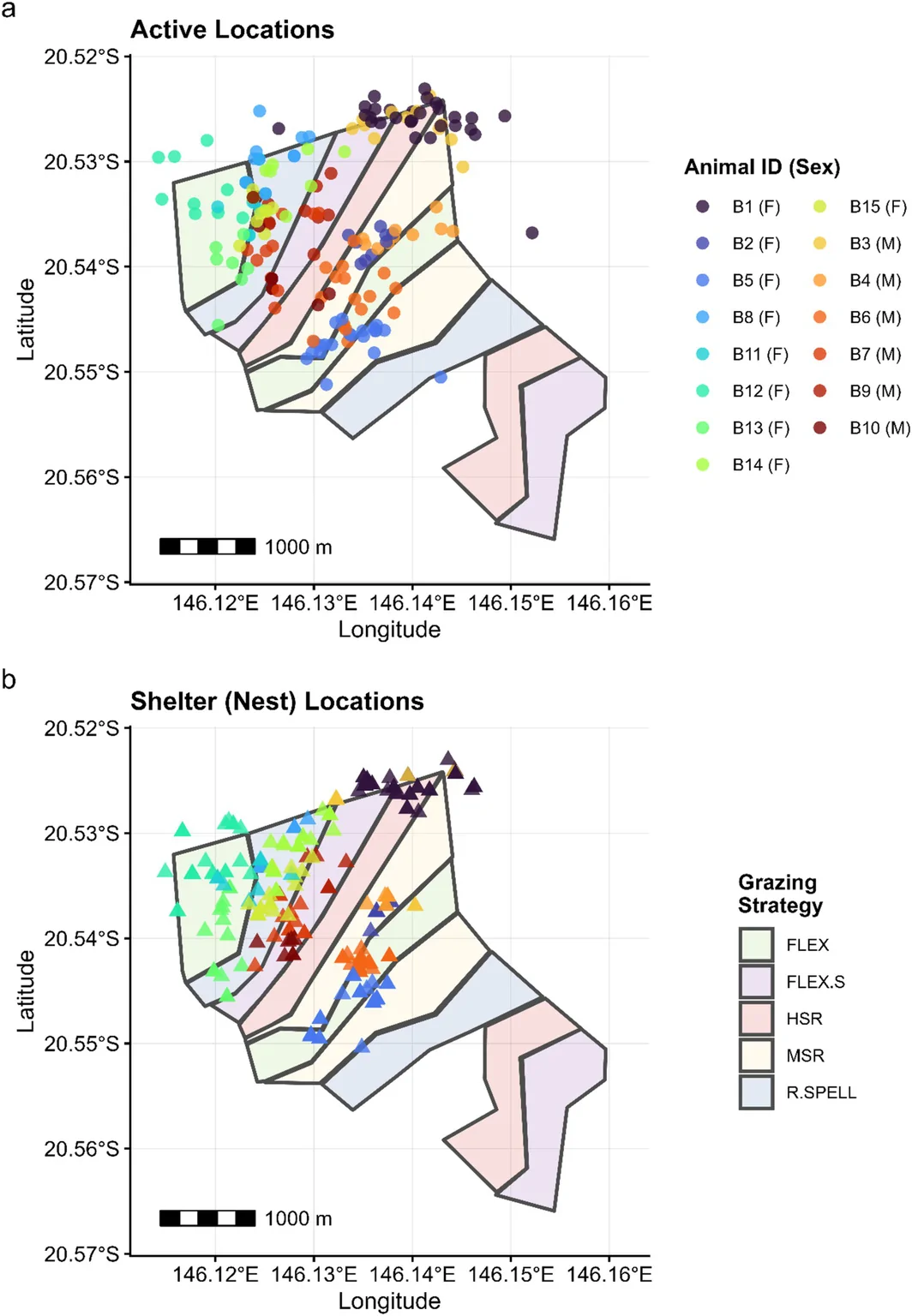
Spatial distribution of rufous bettong ( *Aepyprymnus rufescens* ) locations while active (a; nocturnal activity) and shelter/nest site locations (b; diurnal shelter sites) across grazing management strategies at Wambiana, north Queensland. Points represent individual GPS locations, with circles indicating locations when bettongs were active at night and triangles indicating shelter sites during the day. Colors distinguish individual animals (*n* = 15; nine females, six males). Paddock boundaries delineate five grazing strategies (see Table 1 for descriptions).

Rufous bettongs demonstrated some shelter site fidelity, reusing 27.2% (37/136) of shelter locations. The mean interval between reuse events was 12.2 ± 23.7 days (range: 1–160 days), with most returns occurring within two weeks, suggesting that both short‐term and long‐term site fidelity may influence shelter selection.

### 3.2 Landscape‐Scale Shelter Site Selection and Movement

Animals were tracked for an average of 168 days (range: 22–429 days). The total distance travelled ranged from 4.09 km (B2, female) to 33.06 km (B1, female), with females averaging 12.0 ± 9.0 km and males averaging 9.2 ± 4.4 km over the tracking period. Home range size varied among individuals but showed substantial overlap between sexes (Figure 3; Table 2). Using 100% minimum convex polygons (MCP), home ranges averaged 58.7 ± 56.1 ha for females and 52.7 ± 31.2 ha for males. The 95% kernel density estimates (KDE), representing core use areas, were 112.6 ± 44.3 ha for females and 154.1 ± 104.1 ha for males. Intensive use areas (50% KDE) averaged 30.0 ± 12.2 ha for females and 35.8 ± 25.8 ha for males. Most individuals used paddocks with multiple grazing strategies (females: 2.22 ± 0.67; males: 2.83 ± 0.98), indicating movement across different paddock management types.

**FIGURE 3:**
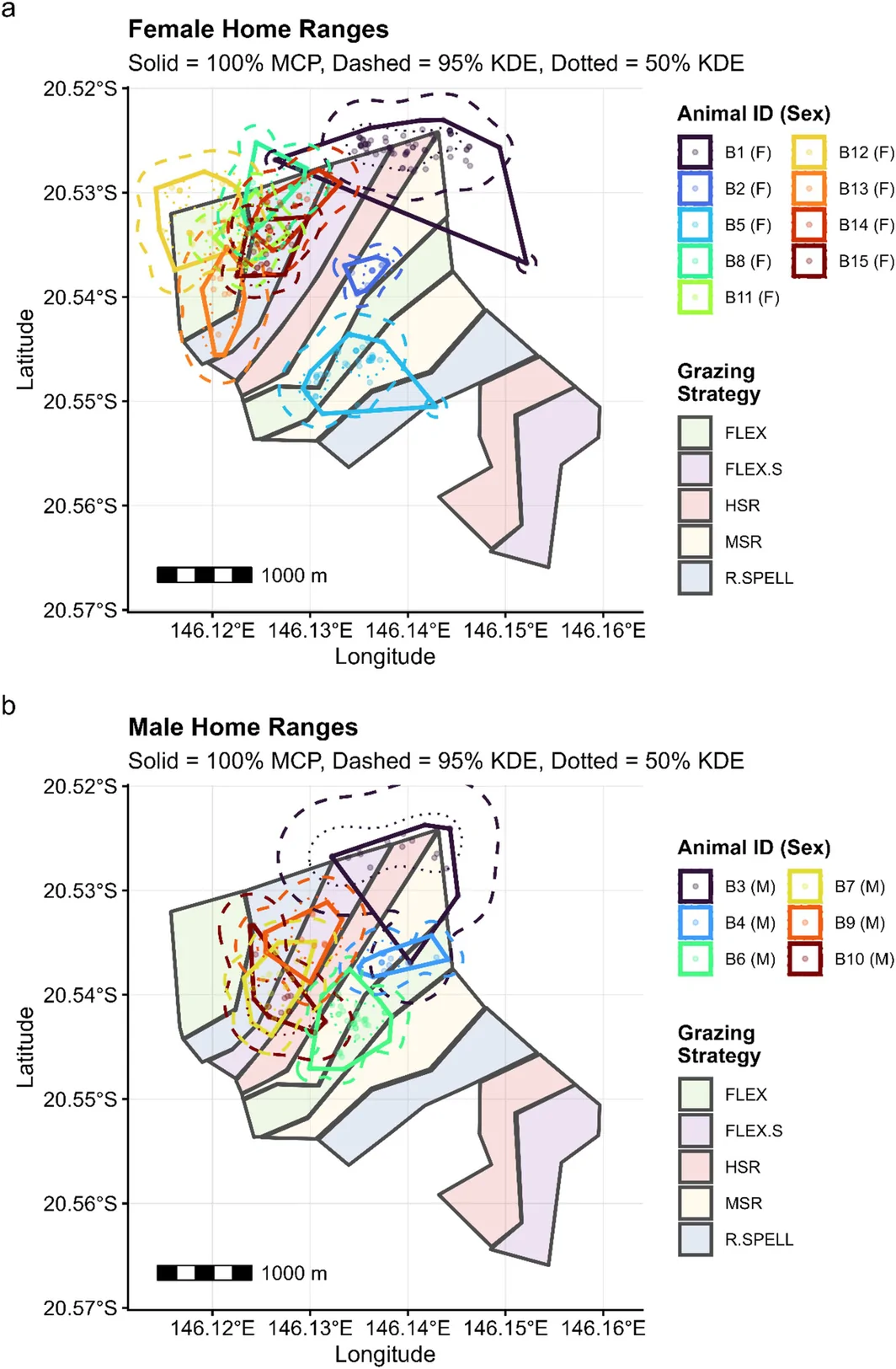
Female (a) and male (b) home range estimates using all locations (active and shelter sites) for 15 rufous bettongs at the Wambiana grazing trial. Solid lines = 100% MCP, dashed lines = 95% KDE (core use area), dotted lines = 50% KDE (intensive use area). Home ranges overlapped extensively across multiple grazing strategies (FLEX, FLEX.S, HSR, MSR, R.SPELL; See Table 1 for descriptions).

**TABLE 2:** Home range size and movement patterns for 15 rufous bettongs ( *Aepyprymnus rufescens* ) at the Wambiana Grazing trial.

Animal ID	Sex	Relocations (N)	MCP 100% (ha)	KDE 95% (ha)	KDE 50% (ha)	Total distance (km)	Strategies used
B1	F	52	197.75	179.50	41.71	33.06	FLEX.S, HSR, MSR
B2	F	13	10.18	31.66	7.92	4.09	HSR, MSR
B5	F	35	74.87	118.04	27.31	18.50	FLEX, MSR
B8	F	16	34.48	111.56	29.25	6.56	FLEX, R.SPELL
B11	F	13	31.36	88.21	24.05	4.76	FLEX, R.SPELL
B12	F	26	73.85	163.29	48.44	10.38	FLEX
B13	F	20	32.68	127.91	37.44	11.38	FLEX, R.SPELL
B14	F	25	39.06	115.96	34.02	11.32	FLEX, FLEX.S, R.SPELL
B15	F	22	33.77	77.63	19.50	7.92	FLEX, FLEX.S, R.SPELL
B3	M	14	110.98	357.61	85.26	16.23	FLEX, FLEX.S, HSR, MSR
B4	M	12	21.05	67.51	12.08	5.61	FLEX, MSR
B6	M	32	60.57	98.70	18.71	12.77	FLEX, HSR, MSR
B7	M	19	44.64	116.77	31.10	8.93	FLEX.S, R.SPELL
B9	M	12	38.63	127.03	34.16	5.17	FLEX.S, R.SPELL
B10	M	13	40.33	156.78	33.47	6.48	FLEX, FLEX.S, MSR, R.SPELL
Mean	F_*n* = 9_	24.7 12.4	58.67	112.64	29.96	12.00	
SD			56.12	44.29	12.19	9.02	
Mean	M_*n* = 6_	17.0 7.8	52.70	154.07	35.80	9.20	
SD			31.23	104.04	25.81	4.44	

*Note:* Home ranges estimated using 100% MCP and 95%/50% KDE. Total distance represents sum of straight‐line movements between locations. Strategies Used indicates number of grazing paddocks used. Animals ordered by sex (Females, then Males) and ID.

Manly's Type II habitat selection analysis revealed significant non‐random habitat selection at the individual level (pooled analysis; *χ* ^2^ = 608.9, df = 60, *p* < 0.001), indicating that individual bettongs did not use grazing strategies in proportion to their availability. However, selection patterns varied substantially among individuals (Table 3), and no population‐level preference or avoidance of any single grazing strategy was detected after Bonferroni correction (all 95% CIs for *W*_i_ overlapped 1; Figure 4). Post hoc Wilcoxon rank‐sum tests revealed no significant differences in selection ratios between sexes for any grazing strategy.

**TABLE 3:** Individual Manly Type II selection ratios (*W*_ij_) for 15 rufous bettongs ( *Aepyprymnus rufescens* ) across five grazing strategies at Wambiana Station, Queensland.

Individual	Sex	HSR	MSR	R.SPELL	FLEX	FLEX.S
B1	F	4.13	0.58	0.00	0.00	1.07
B2	F	0.38	3.85	0.00	0.00	0.00
B5	F	0.00	2.26	0.15	1.64	0.00
B8	F	0.00	0.00	6.27	0.45	0.00
B11	F	0.00	0.00	3.55	1.07	1.11
B12	F	0.00	0.00	0.00	3.34	0.67
B13	F	0.00	0.00	2.84	2.12	0.25
B14	F	0.00	0.00	6.16	0.13	0.50
B15	F	0.00	0.00	5.74	0.45	0.38
B3	M	1.43	0.91	0.00	0.86	1.67
B4	M	0.00	3.31	0.00	0.73	0.00
B6	M	0.18	1.36	0.00	2.46	0.00
B7	M	0.00	0.00	3.23	0.00	2.73
B9	M	0.00	0.00	2.03	0.00	3.57
B10	M	1.21	0.51	1.33	0.24	2.19
Pooled *W*_ᵢ_		0.48	0.96	2.05	0.98	0.74
95% CI lower		−0.40	0.09	0.24	0.25	0.08
95% CI upper		1.35	1.84	3.87	1.70	1.41

*Note:* Selection ratios > 1 indicate disproportionately high use relative to availability (preference), values < 1 indicate disproportionately low use (avoidance), and values of 0 indicate the grazing strategy was not used by that individual. Grazing strategies are ordered from highest to lowest stocking intensity. Pooled population‐level selection ratios (*W*_ᵢ_) with Bonferroni‐corrected 95% confidence intervals are presented in the final rows; no population‐level preference or avoidance was statistically significant (all CIs overlap 1).

Abbreviations: F = female, M = male.

**FIGURE 4:**
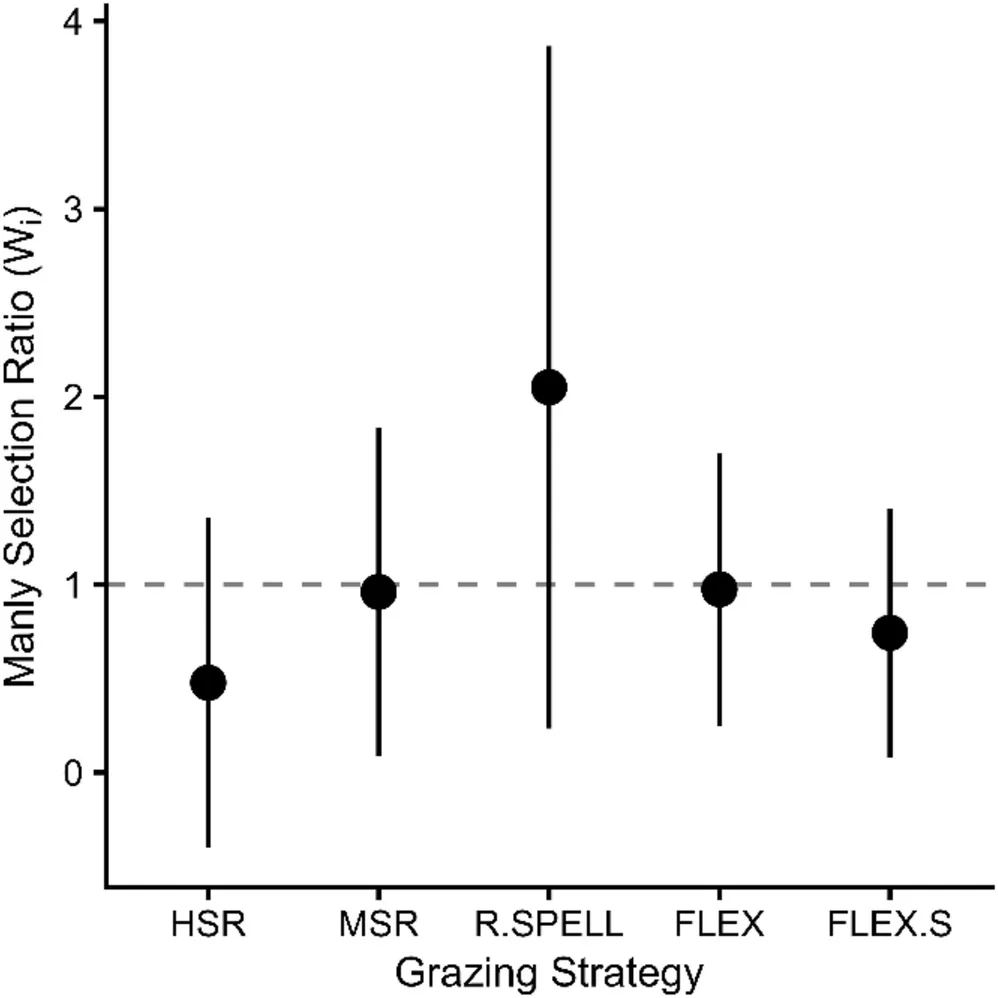
Manly selection ratios (*W*_ᵢ_ ± 95% CI) for shelter site habitat selection by 15 rufous bettongs (pooled analysis). The dashed line indicates proportional use (*W*_ᵢ_ = 1); values above indicate selection, values below indicate avoidance. Confidence intervals are Bonferroni‐corrected.

Despite the lack of statistical significance at the population level, pooled selection ratios suggested a directional trend toward preference for R.SPELL (rotational spelling; *W*_ᵢ_ = 2.05) and avoidance of HSR (heavy stocking rate; *W*_ᵢ_ = 0.48), with near‐proportional use of FLEX (*W*_ᵢ_ = 0.98), MSR (*W*_ᵢ_ = 0.96), and FLEX.S (*W*_ᵢ_ = 0.74).

### 3.3 Fine‐Scale Shelter Site Selection

We surveyed 133 unique shelter sites and 224 random sites across wet and dry seasons (2020–2021). Structural composition differed significantly between shelter and random sites (MANOVA; Pillai's trace = 0.49, *F*_10,344_ = 32.54, *p* < 0.001; Figure 5). Shelter sites were characterized by high proportions of *Carissa ovata* (*F*_1,353_ = 168.9, *p* < 0.001, *η* ^2^_p_ = 0.32), while random sites had higher proportions of bare ground (*F*_1,353_ = 77.1, *p* < 0.001, *η* ^2^_p_ = 0.18), leaf litter (*F*_1,353_ = 65.7, *p* < 0.001, *η* ^2^_p_ = 0.16), and grass (*F*_1,353_ = 38.7, *p* < 0.001, *η* ^2^_p_ = 0.10). Shelter sites also had significantly more logs (*F*_1,353_ = 10.6, *p* = 0.001, *η* ^2^_p_ = 0.03), termite mounds (*F*_1,353_ = 10.1, *p* = 0.002, *η* ^2^_p_ = 0.03), trees (*F*_1,353_ = 8.8, *p* = 0.003, *η* ^2^_p_ = 0.02), and saplings/shrubs (*F*_1,353_ = 5.1, *p* = 0.025, *η* ^2^_p_ = 0.01) than random sites. Fine woody debris (*F*_1,353_ = 1.1, *p* = 0.30) and canopy cover (*F*_1,353_ = 0.4, *p* = 0.55) did not differ between site types.

**FIGURE 5:**
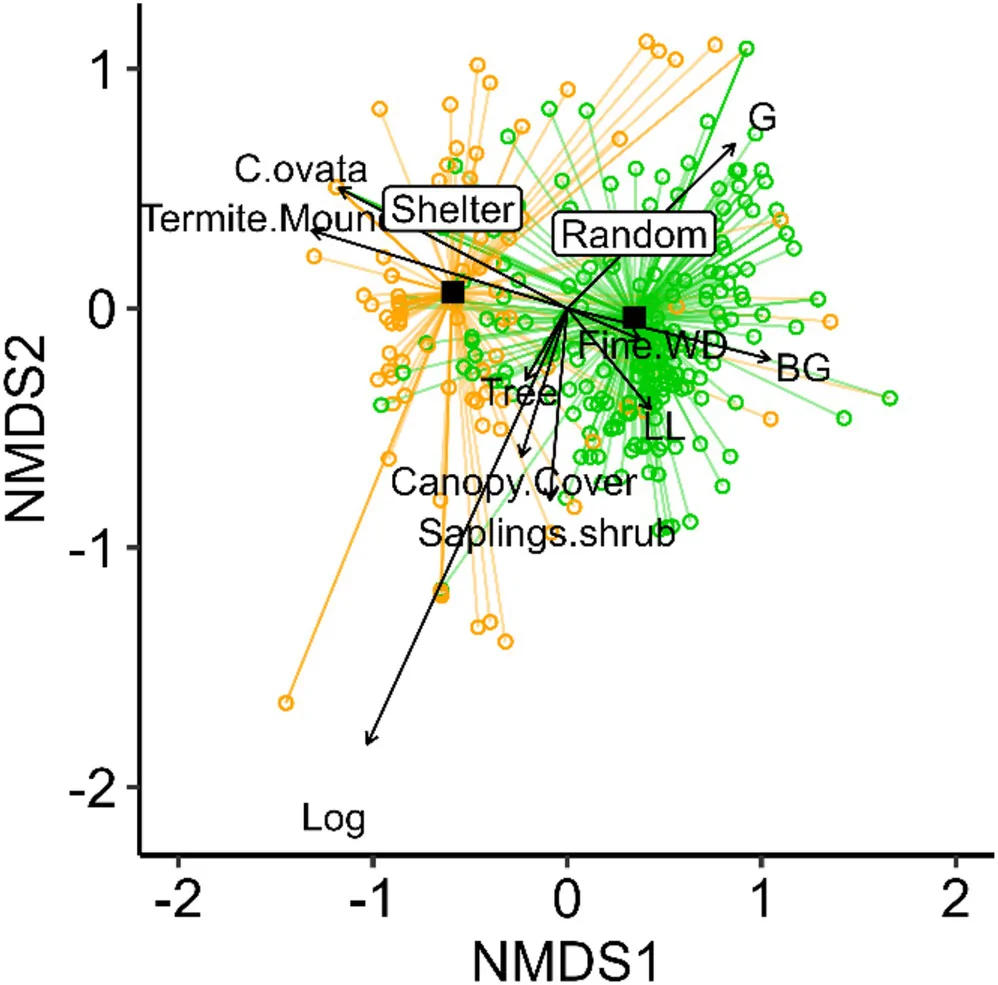
Non‐metric multidimensional scaling (NMDS) ordination of structural habitat composition at rufous bettong shelter sites (orange; *n* = 133) and paired random sites (green; *n* = 224). Points represent individual sites connected to group centroids (black squares) by lines. Vectors indicate the direction and relative strength of association for each structural variable: *Carissa ovata* (*C. ovata*), leaf litter (LL), bare ground (BG), grass (G), logs (Log), fine woody debris (Fine.WD), trees (Tree), saplings/shrubs (Saplings. shrub), termite mounds (Termite. Mound), and canopy cover (Canopy. Cover). Stress = 0.14. This stress value falls within the range generally considered to provide a fair representation of multivariate distances in reduced dimensions (Clarke 1993), though it indicates some distortion; interpretations should therefore be considered alongside the MANOVA results, which do not rely on dimensional reduction.

Rufous bettongs did not shelter exclusively in *C. ovata* ; shelters also occurred in or beside logs, termite mounds, and thick grass, reflecting the multivariate nature of shelter site selection.

### 3.4 Shelter Site Temperatures

We restricted the thermal analysis to daytime hours (0600–1800 h), as this is the period during which rufous bettongs occupy shelters. Across both seasons, we collected daytime temperature readings from iButton data loggers deployed inside shelters and at paired ground‐level points 2 m away (dry season: 10 shelter and 8 unsheltered loggers were recovered; wet season: 8 shelter and 7 unsheltered loggers were recovered).

Shelter sites exhibited substantially reduced thermal variation compared to random sites across both seasons (Figure 6). In the dry season, mean daytime temperatures at shelter sites ranged from 19.6°C to 30.2°C, whereas random sites experienced a wider range (14.1°C to 41.5°C). In the wet season, shelter sites ranged from 27.4°C to 31.6°C compared to 24.6°C to 39.2°C at random sites. Temperature variability (SD) was markedly lower at shelter sites (dry: 5.6°C; wet: 3.0°C) than at random sites (dry: 11.2°C; wet: 7.1°C).

**FIGURE 6:**
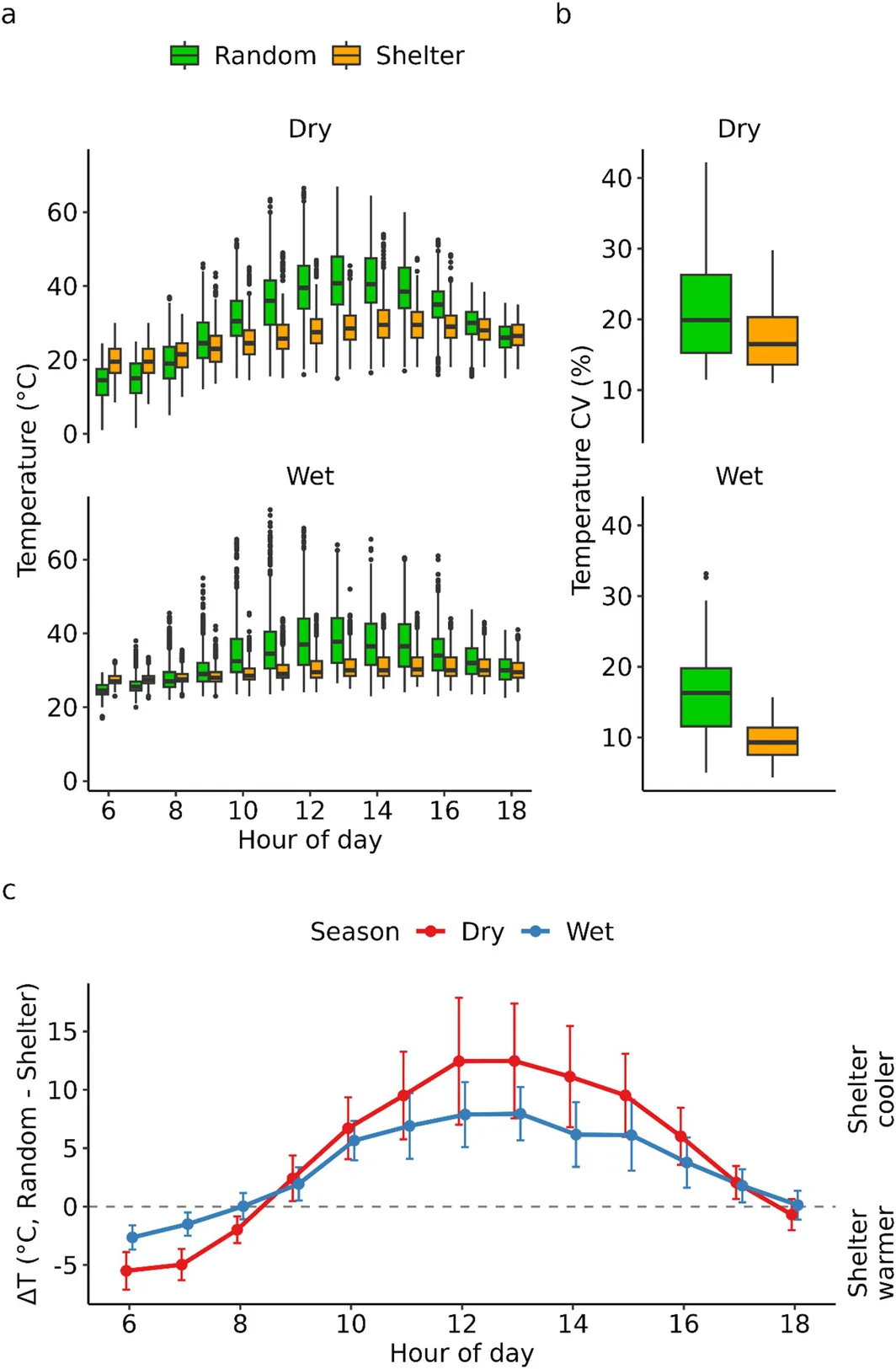
Thermal buffering of rufous bettong shelters compared with paired ground‐level points 2 m from each shelter, during daytime hours (0600–1800 h) in the dry and wet seasons. (a) Hourly temperature distributions inside shelters and at paired unsheltered points. (b) Overall thermal variability, expressed as the coefficient of variation of temperature (CV %); fills as in (a). (c) Mean hourly Δ*T* (unsheltered minus shelter temperature) with 95% confidence intervals; positive values indicate that the shelter was cooler than the unsheltered ground 2 m away, and negative values that it was warmer. Confidence intervals are calculated across loggers, the unit of replication, each of which contributed 90–120 days of hourly readings. Dry season: 10 shelter and 8 unsheltered loggers; wet season: 8 shelter and 7 unsheltered loggers. Boxplots show the median, interquartile range, and outliers. Temperatures above 70°C recorded at unsheltered points during the wet season were verified against the raw logger files and are consistent with ground‐surface temperatures under direct solar radiation in the tropical wet season.

Hourly Δ*T* values (random minus shelter temperature), where positive values indicate that shelters were cooler than random sites, confirmed that shelters were consistently cooler during peak daytime hours. In the dry season, mean Δ*T* was greatest during morning warming (0800–1000 h), when random site temperatures rose rapidly while shelter temperatures remained stable. In the wet season, Δ*T* was greatest during the middle of the day (1000–1500 h), reflecting the pronounced thermal buffering provided by shelters during the hottest part of the day.

To quantify thermal buffering capacity, we modelled the coefficient of variation (CV) of temperature using linear mixed‐effects models with location, hour, and their interaction as fixed effects, and iButton ID as a random effect. The effect of location on thermal variability depended on time of day in both seasons (dry season: Location × Hour *χ* ^2^ = 122.7, df = 23, *p* < 0.001; wet season: Location × Hour *χ* ^2^ = 338.9, df = 23, *p* < 0.001; Figure 6). The Models explained substantial variance in thermal variability (dry season: marginal *R* ^2^ = 0.59, conditional *R* ^2^ = 0.80; wet season: marginal *R* ^2^ = 0.87, conditional *R* ^2^ = 0.89). In the dry season, random sites showed a substantially higher CV than shelter sites throughout the day, with the greatest differences occurring during morning hours (0600–0900) when random site temperatures were rapidly warming while shelter temperatures remained stable. In the wet season, both location types showed low CV during early morning but diverged markedly during midday (0900–1700), with random sites exhibiting 2–3 times greater thermal variability than shelters (Figure 6).

Thermal buffering was not restricted to shelters in shaded situations. Daytime maximum temperatures inside shelters spanned 31.0°C–54.0°C in the dry season and 37.5°C–52.0°C in the wet season, indicating that shelter sites encompassed a wide range of solar exposure. Despite this range, every shelter logger recorded lower daytime thermal variability than every paired unsheltered logger in both seasons (dry season: shelter CV 14.7%–31.3%, unsheltered 31.8%–44.0%, Mann–Whitney *U* = 0, *p* < 0.001; wet season: shelter CV 8.3%–13.3%, unsheltered 21.2%–27.3%, *U* = 0, *p* < 0.001), with no overlap between the two distributions in either season. The most exposed shelter recorded in the dry season reached 54.0°C yet still showed lower thermal variability (CV 31.3%) than the least variable unsheltered point (31.8%).

## 4 Discussion

Rufous bettong shelters provided substantial thermal buffering, reducing daytime temperature extremes by up to 21.5°C relative to unsheltered ground 2 m away. This finding suggests that shelters are critical thermal refugia for medium‐sized mammals in hot environments. At the fine scale, bettongs selected shelter sites with high structural complexity, particularly *Carissa ovata* thickets, logs, and termite mounds. The grazing strategy did not significantly affect shelter site selection, though bettongs tended to avoid heavily grazed paddocks.

### 4.1 Landscape‐Scale Shelter Site Selection

Home range sizes in our study (MCP: 58.7 ha for females, 52.7 ha for males; 95% KDE: 112.6 ha for females, 154.1 ha for males) were broadly comparable to previous estimates for rufous bettongs. Frederick and Johnson (1996) reported home ranges of 30–60 ha using MCP, while Johnson et al. (2005) reported 95% KDE estimates of 50–120 ha. The larger KDE estimates in our study may reflect the open, grazed landscape at Wambiana, where bettongs ranged across multiple paddocks. Home range size did not differ significantly between sexes, consistent with previous findings for this species.

Individual rufous bettongs ranged across multiple grazing strategies, suggesting that they perceived the trial as a mosaic of grazing intensities rather than discrete habitats. Paddock boundaries consisted of minimal three‐strand non‐barbed wire fences (middle strand electrified), which do not impede bettong movement; animals moved freely beneath the lowest wire between paddocks. Fence lines were not associated with additional vegetation or structural complexity.

Pooled selection ratios indicated a trend for bettongs to prefer the rotational spelling paddocks while avoiding heavily grazed paddocks, though these patterns were not statistically significant after Bonferroni correction. The directional trends are ecologically coherent: heavy continuous grazing reduces the structural complexity of ground‐layer vegetation (Neilly, O'Reagain, et al. 2018), which many small‐ and medium‐sized mammals rely on for predator avoidance and shelter (Fischer et al. 2004; Lunt et al. 2007). The limited sample size of tracked individuals (*n* = 15) reduced the statistical power to detect significant selection after correction for multiple comparisons, and these trends warrant further investigation with larger sample sizes. Similar patterns have been documented in other ground‐dwelling mammals; northern brown bandicoots ( *Isoodon macrourus* ) show reduced activity in heavily grazed areas where understorey cover is depleted (Legge et al. 2011).

Previous studies at Wambiana found stronger avoidance of heavy grazing strategies during drought years, when contrasts in land condition between strategies were more pronounced (Neilly and Schwarzkopf 2018; O'Reagain et al. 2018). Our study was conducted during moderately wet climatic conditions; post‐dry season pasture yields at Wambiana averaged 201–397 kg/ha compared to 639–774 kg/ha post‐wet season (O'Reagain et al. 2018), and the relatively high pasture availability during our study period likely reduced structural contrasts among grazing treatments. This may have masked stronger selection patterns that emerge during drought, when differences in land condition between strategies are more pronounced. Longer‐term studies incorporating both wet and drought periods would clarify whether rufous bettongs consistently avoid heavily grazed areas and how climate variability influences shelter site selection. The spatial concentration of shelters in the northern paddocks likely reflects the higher availability of *Carissa ovata* in Reid River box (*Eucalyptus brownii*) woodland (Neilly and Schwarzkopf 2017), which was present at 84.6% of shelter sites (115 of 136), rather than a response to grazing strategy per se.

### 4.2 Fine‐Scale Shelter Site Selection

Rufous bettongs selected shelter sites with a high proportion of *Carissa ovata*, representing the largest effect size among all habitat variables. This native shrub has leathery leaves and thorny stems, often forming dense thickets several meters tall and wide (Department of Agriculture and Fisheries 2020). These thickets are nearly impenetrable, providing excellent refuge for small mammals (Christensen and Leftwich 1980; Fisher 2000; Poole et al. 2015). *Carissa ovata* likely provides concealment and protection from predators (Day and Elwood 1999; Fisher 2000), including dingoes, wild dogs, and feral cats, as well as protection from cattle trampling, which has been identified as a potential factor influencing habitat selection in this species (Neilly and Schwarzkopf 2018).

Bettongs used *Carissa ovata* as shelter more frequently in the dry season, reflecting reduced grass availability following the dry season. Because rufous bettongs rely on grass to construct their shelters (Schlager 1982), reduced grass availability may increase reliance on *C. ovata* for adequate shelter. We also observed repeated reuse of shelter sites, which may become more common during very dry years when grass cover is particularly low, as a strategy to avoid the energetic costs of constructing new shelters. Shelter construction requires time and energy to locate, transport, and assemble nesting material (Geiser and Ferguson 2001), and sheltering within existing shrub thickets may reduce these costs by minimizing time spent searching for materials. However, our study was conducted during a relatively wet year with abundant grass resources, so this pattern may not have been fully expressed. As *C. ovata* was the dominant shrub at our site, we could not determine whether bettongs specifically select this species or opportunistically use available dense shrubs. Other small‐ and medium‐sized mammals, including quokkas and wallabies, also preferentially shelter in dense shrub thickets (Fisher 2000; Poole et al. 2015).

In the wet season, bettongs increased their use of logs and termite mounds, which may provide greater thermal buffering than *C. ovata* thickets. Termite mounds and hollow logs offer more stable microclimates than grass shelters, due to their thermal mass and insulation properties (Korb 2003). Increased use of solid structures during the wet season may also reduce the risk of shelter flooding during torrential rains and provide greater thermal buffering during summer temperature extremes. Canopy cover did not differ between shelter and random sites, suggesting that overhead tree cover is not a primary driver of shelter site selection in this species. Bettongs may prioritize ground‐level concealment and structural complexity over canopy shading.

### 4.3 Shelter Site Temperatures

The thermal buffering by shelter sites was greatest during midday hours and consistent across seasons. As rufous bettongs are nocturnal and occupy shelters throughout the day, this thermal buffering is critical for survival by mitigating high daytime temperatures. Exposure to the extreme surface temperatures recorded at random sites (up to 67°C in the dry season and 73.5°C in the wet season) would be lethal with prolonged exposure. The upper critical temperature (*T*_uc_) for *Aepyprymnus rufescens* is 33°C (Rübsamen et al. 1983; reported in Riek and Geiser 2013, where the species was flagged as an outlier for its unusually low *T*_uc_ among marsupials). Surface temperatures at random sites exceeded the *T*_uc_ by more than 34°C, strongly supporting the assertion that sustained exposure would be fatal. A thermoneutral zone (TNZ) has not been published for the rufous bettong; the closest available data are from the Tasmanian bettong ( *Bettongia gaimardi* ), for which Rose et al. (1990) reported that basal body temperature (36.6°C) showed little variation between ambient temperatures of 5°C–30°C. As *B. gaimardi* occupies a temperate climate, direct extrapolation to rufous bettongs in tropical savanna should be made cautiously, and the determination of the TNZ for *A. rufescens* remains a priority.

Because shelters and their paired thermal controls were separated by only 2 m, the buffering we report cannot be attributed to differences in overstorey shading. Consistent with this, canopy cover did not differ between shelter and random sites (*F*_1,353_ = 0.4, *p* = 0.55), and overhead canopy was entirely absent at 42.7% of shelter sites. Rufous bettongs frequently construct shelters in open situations rather than selecting already‐shaded locations, so the thermal benefit we quantify derives from the constructed structure and its immediate ground layer rather than from overhead shade. We note, however, that our loggers were not stratified by microhabitat and cannot be matched retrospectively to the fine‐scale vegetation plots. We therefore cannot compare a constructed shelter with the best naturally shaded resting site an animal might otherwise have used. Determining whether construction confers a thermal advantage over the best available natural refuge, and comparing the buffering capacity among shelter types ( *C. ovata* thickets, logs and termite mounds), are priorities for targeted future work.

Thermal refugia from temperature extremes are increasingly recognized as critical for wildlife persistence, particularly for species with limited capacity for behavioural thermoregulation during inactive periods (Kerth et al. 2001; Isaac et al. 2008; Hovick et al. 2014). The thermal buffering by shelters is well documented in small mammals, where even modest reductions in temperature variability can substantially reduce thermoregulatory costs (Mathias et al. 2004; McComb et al. 2022; Waudby and Petit 2017; O'Connell and Keppel 2016).

Greater temperature variability at shelter sites during the dry season may reflect differences in shelter construction. The dry season coincides with lowest grass availability (P. O'Reagain, pers. comm 2022), potentially resulting in thinner grass “roofs” with reduced insulation and increased reliance on *C. ovata* thickets, which likely provide less thermal buffering than solid structures. In the wet season, increased use of logs (which provide greater thermal mass and reduce exposure to direct solar radiation and soil surface temperatures; Webb and Shine 1998) may explain the lower temperature variability recorded. Similar patterns have been documented in other Australian mammals, where tree hollows and burrows provide substantial thermal buffering compared to exposed sites (Körtner and Geiser 2000; Waudby and Petit 2017). A targeted comparison of thermal buffering capacity among specific shelter types (e.g., *C. ovata* thickets, logs, termite mounds) is a priority for future research; our study design did not pair thermal loggers with fine‐scale habitat assessments at the same shelter sites, precluding this analysis retrospectively.

### 4.4 Conservation Implications and Management Recommendations

Conserving rufous bettongs on grazed land should be a conservation priority due to their role as ecosystem engineers providing benefits to soil function and other animal and plant species (H. Neilly, N. Ryan, E. Nordberg, and L. Schwarzkopf, unpublished data). Management that maintains diverse shelter resources, shrub thickets, logs, termite mounds, and adequate grass for shelter construction—will support rufous bettong populations and likely benefit other ground‐dwelling mammals sharing these habitats. Grazing strategies that incorporate rotational spelling may benefit bettongs by maintaining ground‐layer vegetation structure needed for shelter construction and concealment. Heavy continuous grazing reduces structural complexity and may limit shelter availability during drought, when bettongs are most vulnerable (Legge et al. 2011; Neilly and Schwarzkopf 2017). As climate change increases the frequency and intensity of temperature extremes across northern Australia (Hughes 2003), thermal refugia will become increasingly important for wildlife persistence.

## 5 Conclusions

Rufous bettong shelters function as critical thermal refugia in a hot, seasonally variable environment. Constructed shelters reduced daytime temperature extremes by over 20°C relative to unsheltered ground 2 m away, and thermal buffering was most pronounced during the middle of the day when surface temperatures on unsheltered ground exceeded 70°C. The upper critical temperature for this species (33°C; Rübsamen et al. 1983) is far below the temperatures recorded on unsheltered ground, underscoring the essential role of shelter construction for survival. Shelter site selection was driven primarily by fine‐scale habitat features, particularly *Carissa ovata* thickets, logs, and termite mounds, rather than by landscape‐scale grazing management. These findings highlight that retention of structurally complex ground‐layer habitat, including dense shrub thickets, coarse woody debris, and adequate grass cover for shelter construction, is essential for rufous bettong persistence in grazed landscapes under current and future climate conditions.

## Author Contributions

**Eric J. Nordberg:** conceptualization (equal), data curation (supporting), formal analysis (equal), investigation (supporting), methodology (equal), project administration (equal), supervision (supporting), validation (equal), visualization (equal), writing – original draft (equal), writing – review and editing (equal). **Natasha Ryan:** data curation (lead), formal analysis (equal), investigation (lead), methodology (equal), project administration (equal), validation (equal), visualization (equal), writing – original draft (equal), writing – review and editing (equal). **Heather Neilly:** conceptualization (equal), methodology (equal), project administration (equal), supervision (supporting), writing – original draft (supporting), writing – review and editing (equal). **Lin Schwarzkopf:** conceptualization (equal), funding acquisition (lead), project administration (equal), resources (lead), supervision (lead), writing – original draft (supporting), writing – review and editing (equal).

## Funding

The Wambiana grazing trial is co‐funded by the Queensland Department of Agriculture and Fisheries and Meat and Livestock Australia. HN is funded through an ARC Early Career Industry Fellowship.

## Conflicts of Interest

The authors declare no conflicts of interest.

## Supporting information


**Video S1:** Rufous bettong nest building behaviour. Video by Eric Nordberg.

## Data Availability

The data that support the findings of this study are available from https://doi.org/10.6084/m9.figshare.31770166.
